# Probing the Gas-Phase Dynamics of Graphene Chemical Vapour Deposition using *in-situ* UV Absorption Spectroscopy

**DOI:** 10.1038/s41598-017-06276-y

**Published:** 2017-07-21

**Authors:** Abhay Shivayogimath, David Mackenzie, Birong Luo, Ole Hansen, Peter Bøggild, Timothy J. Booth

**Affiliations:** 1 DTU Nanotech, Technical University of Denmark, Ørsteds Plads, 345E, Kongens Lyngby, 2800 Denmark; 2 Centre for Nanostructured Graphene (CNG), Technical University of Denmark, Ørsteds Plads, 345C, Kongens Lyngby, 2800 Denmark

## Abstract

The processes governing multilayer nucleation in the chemical vapour deposition (CVD) of graphene are important for obtaining high-quality monolayer sheets, but remain poorly understood. Here we show that higher-order carbon species in the gas-phase play a major role in multilayer nucleation, through the use of *in-situ* ultraviolet (UV) absorption spectroscopy. These species are the volatilized products of reactions between hydrogen and carbon contaminants that have backstreamed into the reaction chamber from downstream system components. Consequently, we observe a dramatic suppression of multilayer nucleation when backstreaming is suppressed. These results point to an important and previously undescribed mechanism for multilayer nucleation, wherein higher-order gas-phase carbon species play an integral role. Our work highlights the importance of gas-phase dynamics in understanding the overall mechanism of graphene growth.

## Introduction

Large-area, high quality monolayer graphene suitable for applications in flexible electronics can now be reproducibly obtained by chemical vapour deposition (CVD) of graphene on copper, thanks to extensive research into understanding its growth process over the past years^1–5^. It is generally agreed that the growth process begins with an influx of carbon precursors onto the copper surface, which leads to a local supersaturation of active carbon species and triggers the nucleation of graphene domains^6, 7^. The growth of these nascent nuclei depletes the carbon concentration in their vicinity, which suppresses further nucleation^7, 8^. The nuclei continue to expand and eventually coalesce to form extended sheets that span the entire surface of the substrate. Due to the limited solubility of carbon in copper, the reaction is surface-limited, thereby providing a high selectivity towards monolayer growth^9^. The dynamics of the growth process are influenced by four keys parameters: temperature, pressure, methane (CH_4_) concentration, and hydrogen (H_2_) concentration. The effects of temperature are well-understood: higher temperatures generally lead to higher quality films by improving the mobility and adsorption-desorption kinetics of surface-bound carbon, thereby improving the grain crystal quality^7, 8^. The system pressure and flowrates govern the mass transport^10^ and residence time of the gases in the chamber^11^, as well as the surface dynamics of the copper substrate on account of copper sublimation at reduced pressures^12, 13^. The effects of varying the methane and hydrogen partial pressures are closely intertwined with the system pressure, with low CH_4_/H_2_ and high CH_4_/H_2_ ratios favouring monolayer growths in the atmospheric pressure and low pressure regimes, respectively^6, 10, 13–16^. The partial pressures of the gases also determine the chemical potential for carbon deposition, and hence the kinetics of graphene growth on the copper surface. A higher methane partial pressure increases the chemical potential, shifting the graphene grain morphology towards diffusion-limited dendritic growth, whereas hydrogen reduces the potential, favouring kinetically-limited growth of hexagonal domains at high partial pressures^3, 17–23^.

However, certain aspects of the role of hydrogen still remain unclear. In particular, while a large hydrogen partial pressure is crucial for obtaining high-quality, hexagonal domains, several studies have reported an increase in multilayer nucleation under such conditions^16, 23–26^. These observations have so far been explained by hydrogenation of the domain edges, which decouples them from the copper surface and allows diffusion of carbon species underneath the initial monolayer^22, 27, 28^.

Here, through the use of a novel *in-situ* gas-phase ultraviolet (UV) absorption spectroscopic technique, we provide evidence for an alternate, possibly complementary explanation, wherein large concentrations of hydrogen activate carbon contaminants in the reaction chamber, volatilizing them and making them available for nucleation. These contaminants, which comprise oils, greases, and condensed products of pyrolysis, accumulate in the chamber as a result of backstreaming from downstream system components, such as the exhaust lines, the vacuum pump, and the foreline trap. The resultant cracking of these species by hydrogen at high temperatures produces the necessary gas-phase precursors for multilayer nucleation. A dramatic reduction in multilayer nucleation is consequently achieved by suppressing backstreaming in a cleaned reaction chamber, which strongly supports this interpretation. The results thus demonstrate that multilayer nucleation can in some cases be wholly ascribed to the volatilized products of reactions between backstreamed contaminants and H_2_, highlighting the importance of the role of gas-phase carbon species in multilayer nucleation.

## Results

### *In-situ* UV spectroscopic measurements

**Figure 1:**
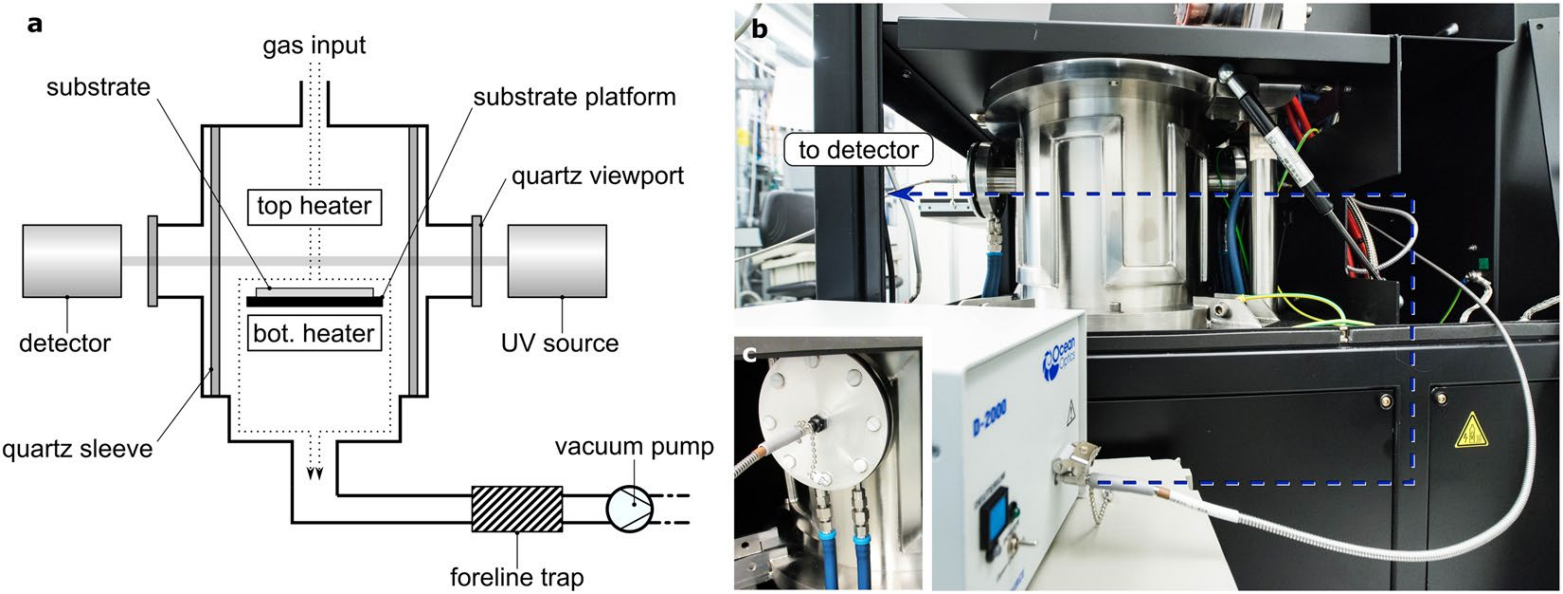
Illustration of the experimental setup. (**a**) schematic of the experimental setup, where a UV spectrometer is mounted on the cold wall CVD system so as to monitor the composition of the gases in the region directly above the copper catalyst layer; (**b**,**c**) photographs of the experimental setup; (**b**) fiber optic cables transmit light (blue dashed line) to and from the detector and UV lamp through the reaction chamber; the cables are attached to collimating lenses, which are firmly and precisely mounted onto the (**c**) front and back viewports.

*In-situ* UV absorption spectroscopy measurements were conducted in a cold-wall CVD system according to Fig. 1. Spectral measurements were continuously recorded from the end of the annealing phase to the end of the growth phase, and the absorbance at each time interval was calculated using the first spectrum in the series as the reference (see Methods). The layout of the system provides direct optical access to the gas directly above the growth substrate. The choice of UV absorption spectroscopy over more conventional infrared and laser-based spectroscopic methods (e.g. - FTIR, coherent anti-stokes Raman spectroscopy, laser-induced fluorescence spectroscopy, cavity ring-down spectroscopy) was made for several reasons. The large infrared background noise from the graphite heaters at typical growth temperatures precludes the use of infrared spectroscopy^29^, whereas laser-based methods run the risk of inducing photochemical reactions and producing spurious results at high temperatures. In contrast, UV absorption spectroscopy is a simple, non-invasive, and sensitive tool that provides direct, real-time measurements of changes in concentrations of photoactive species and is limited largely by instrumental noise^30^.

### *In-situ* spectroscopy and characterization of a standard growth process

**Figure 2:**
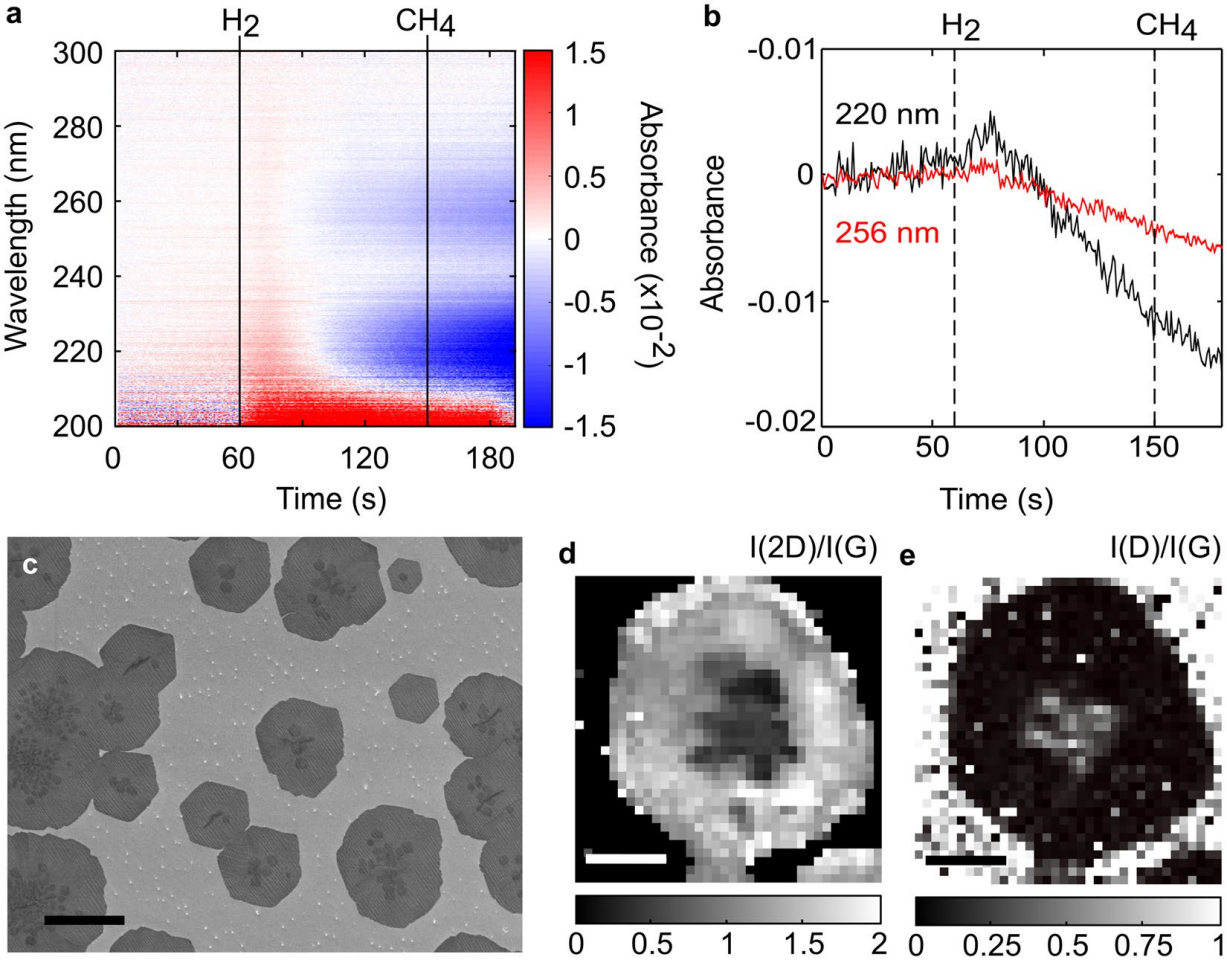
*In-situ* UV absorption spectroscopy and characterization of a standard CVD growth. **(a)** Time evolution of the absorbance spectra and **(b)** line profiles of the 220 nm (black) and 256 nm absorption peaks (red), with the dotted lines at 60 s and 150 s denoting when H_2_ and CH_4_ are introduced, respectively. (**c**) SEM image of the corresponding growth (scale bar 10 µm). (**d,e**) Raman maps of a single graphene grain on copper (**d**) I(2D)/I(G) ratio and (**e**) I(D)/I(G) ratio. Scale bars in (**d**) and (**e**) are 5 µm.

Figure 2 shows the absorption spectra and characterization results of a typical growth on copper foil at 1020 °C, 20 mbar, 1 sccm CH_4_ and 1000 sccm H_2_ flow. High-purity copper foils were fabricated in-house in order to minimize impurities and surface imperfections, which could otherwise adversely influence our results^31^ (see Methods). The sample was annealed at growth temperature in 700 sccm argon (Ar) flow for 30 minutes, after which Ar was turned off and H_2_ was introduced. Following a stabilization period of 90 seconds, the growth phase was executed for 30 seconds by introducing CH_4_.

The addition of hydrogen after 60 seconds is accompanied by an abrupt overall increase in the absorbance, followed by a decrease in the absorbance in two broad bands around 220 nm and 256 nm (Fig. 2a,b) that eventually become negative after approximately 25 seconds. In addition, the 220 nm peak decreases faster than the 256 nm peak. The introduction of CH_4_ after 150 seconds has a negligible effect on the absorption spectrum. Subsequent scanning electron microscopy (SEM) of the copper surface after growth (Fig. 2c) indicates that the graphene grains are monolayers with multilayer regions near the centre of individual domains. Micro-Raman maps (Fig. 2d,e) show I(2D)/I(G) ≈ 2 and I(D)/I(G) ≈ 0 in the peripheral regions of the grain, indicating the presence of defect-free monolayer graphene, and I(2D)/I(G) < 1 and I(D)/I(G) > 0 in the centre of the grain, indicating the presence of a mixture of multi-layered and defective graphene. Some of the grains in Fig. 2c display a hexagonal morphology; however the larger grains are predominantly rounded or irregular with multilayer patches. Moreover, multilayer patches are associated with rounded/irregular grain boundaries in the image, while grains with limited multilayer growth appear more hexagonal.

### Control measurements of the absorption of source gases

**Figure 3:**
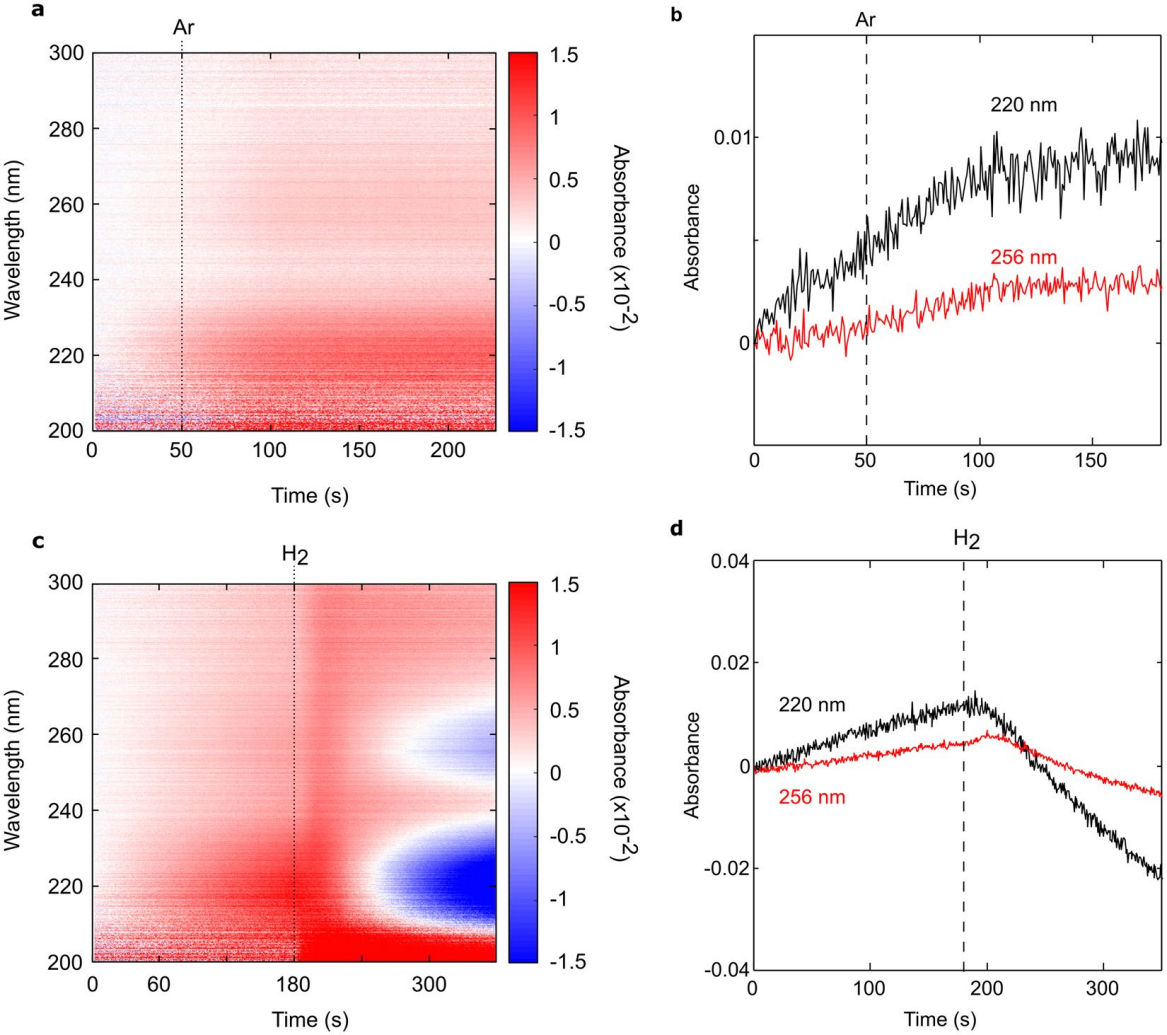
Absorbance spectra of pure argon and hydrogen at growth temperatures. (**a**) Time evolution of the absorbance and (**b**) line profiles of the 220 nm and 256 nm peaks for hydrogen, where the dotted line at 50 s denotes when hydrogen is added in vacuum; (**c**) Time evolution of the absorbance and (**d**) line profiles of the 220 nm and 256 nm peaks for Argon, where the line at 180 s denotes when argon is added in vacuum.

Control measurements of the room temperature absorbance spectra of all source gases, recorded in the absence of a copper substrate in the chamber, show no measureable absorbance relative to vacuum in the spectral regions of interest (Supplementary Fig. 1), confirming that neither the source gases nor any impurities present are responsible for the observed absorption (at least at room temperature). High temperature absorbance measurements of Ar and H_2_ are presented in Fig. 3, and were also recorded in the absence of a copper substrate in the chamber. Prior to adding Ar or H_2_, an initial increase in absorbance at 220 nm (and to a lesser extent at 256 nm) is observed while the chamber is still in vacuum. Adding argon results in a mild increase in absorption across the spectrum (Fig. 3a,b), but in the case of hydrogen the absorption increases initially, and then drops in the same 220 nm and 256 nm spectral regions (Fig. 3c,d) as seen in the standard growth recipe (Fig. 2a). As such, we attribute the absorption behaviour observed in standard growth recipe entirely to the effects of hydrogen at high temperatures.

The Beer-Lambert law states that the absorbance *A*_*i*_ due to an absorbing species *i* is proportional to its concentration *c*_*i*_, its absorption coefficient *ε*_*i*_, and the optical path length *l*:

1 $${A}_{i}={\varepsilon }_{i}{c}_{i}l$$

Changes in absorbance can thus be directly ascribed to changes in concentration of the absorbing species in this experiment. Moreover, a negative absorbance implies that the concentration of said species is lower than when the reference spectrum was recorded.

Our observations therefore suggest that absorbing species accumulate within the chamber when the system is in vacuum, and are gradually removed when hydrogen is introduced. The species responsible for absorption at 220 nm are removed faster than those responsible for the 256 nm absorption. Pressurization of the chamber with argon stabilizes the concentration of said species. Additional experiments where the chamber was cyclically filled with hydrogen and pumped down to vacuum demonstrated the same behaviours, and also resulted in an improvement in base pressure from 0.05 mbar prior to the first hydrogen cycle to 0.038 mbar after the final hydrogen cycle (Supplementary Fig. 2). Moreover, some of the spectral attenuations in vacuum survived upon cooldown to room temperature, but were reversed upon manually cleaning the quartz walls of the reactor (Supplementary Fig. 3). These results cumulatively suggest that hydrogen cleans the chamber of contaminants, which are subsequently re-introduced and accumulate in the chamber when the system is pumped down to vacuum. The contaminants therefore most likely originate from downstream systems components, and diffuse back into the chamber during pumping.

### Absorption measurements of H_2_ and Ar post-cleaning

**Figure 4:**
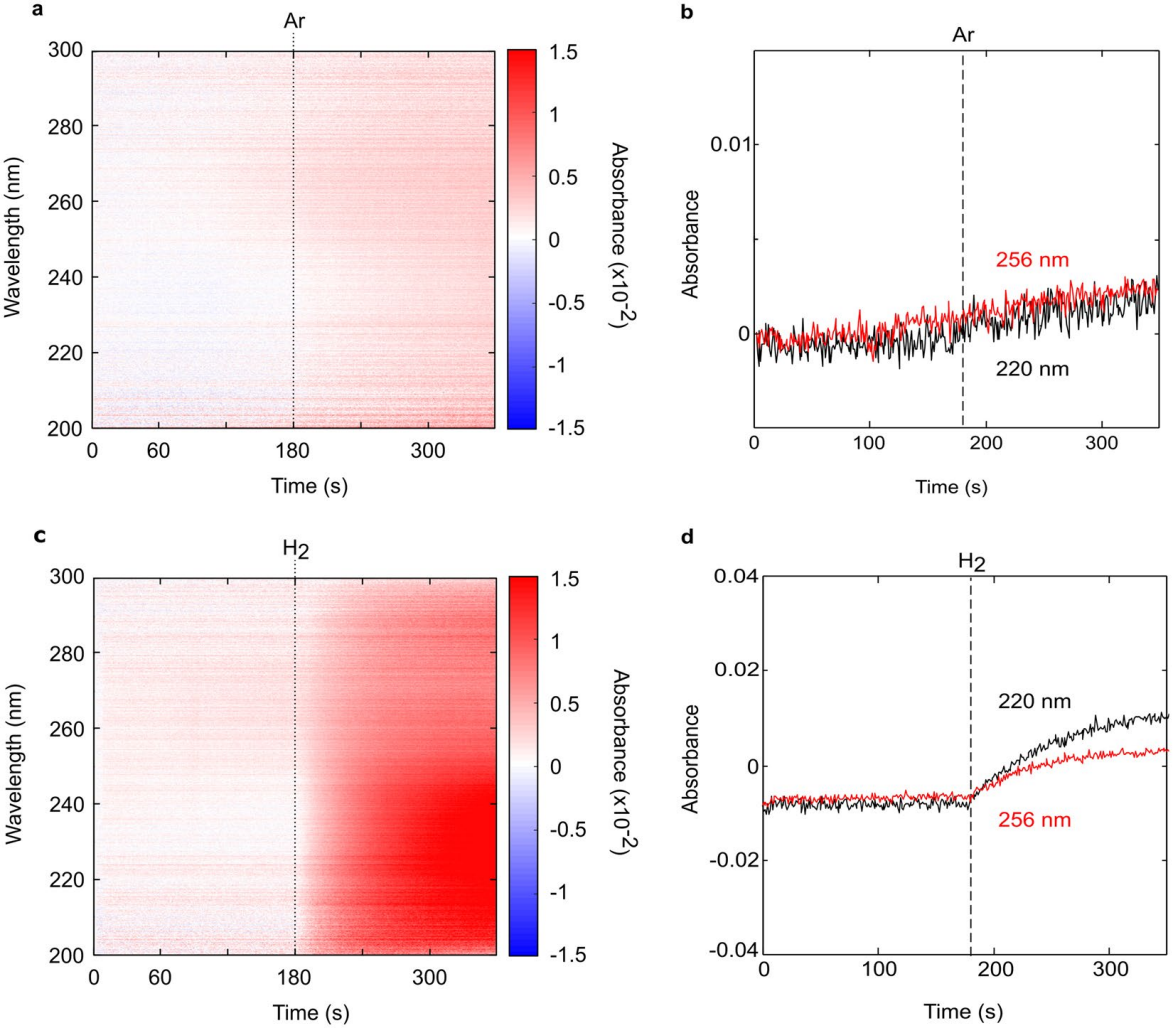
High temperature absorbance spectra of argon and hydrogen post-cleaning. (**a**) Time evolution of the absorbance and (**b**) line profiles of the 220 nm and 256 nm peaks for hydrogen; (**c**) Time evolution of the absorbance and (**d**) line profiles of the 220 nm and 256 nm peaks for Argon. Lines at 180 s denote when the respective gases are added in vacuum.

In light of these findings, we repeated our high temperature Ar and H_2_ absorption control experiments (Fig. 4) after the chamber and downstream components were thoroughly cleaned and/or replaced with new components (see Methods). Introducing Ar (Fig. 4a,b) or H_2_ (Fig. 4c,d) into the chamber now results in a largely uniform attenuation of the spectrum, with no negative absorption peaks previously observed for hydrogen. A broad absorption below 260 nm is now observed in the hydrogen control experiment (Fig. 4c).

### *In-situ* spectroscopy and characterization of a standard growth process post-cleaning

**Figure 5:**
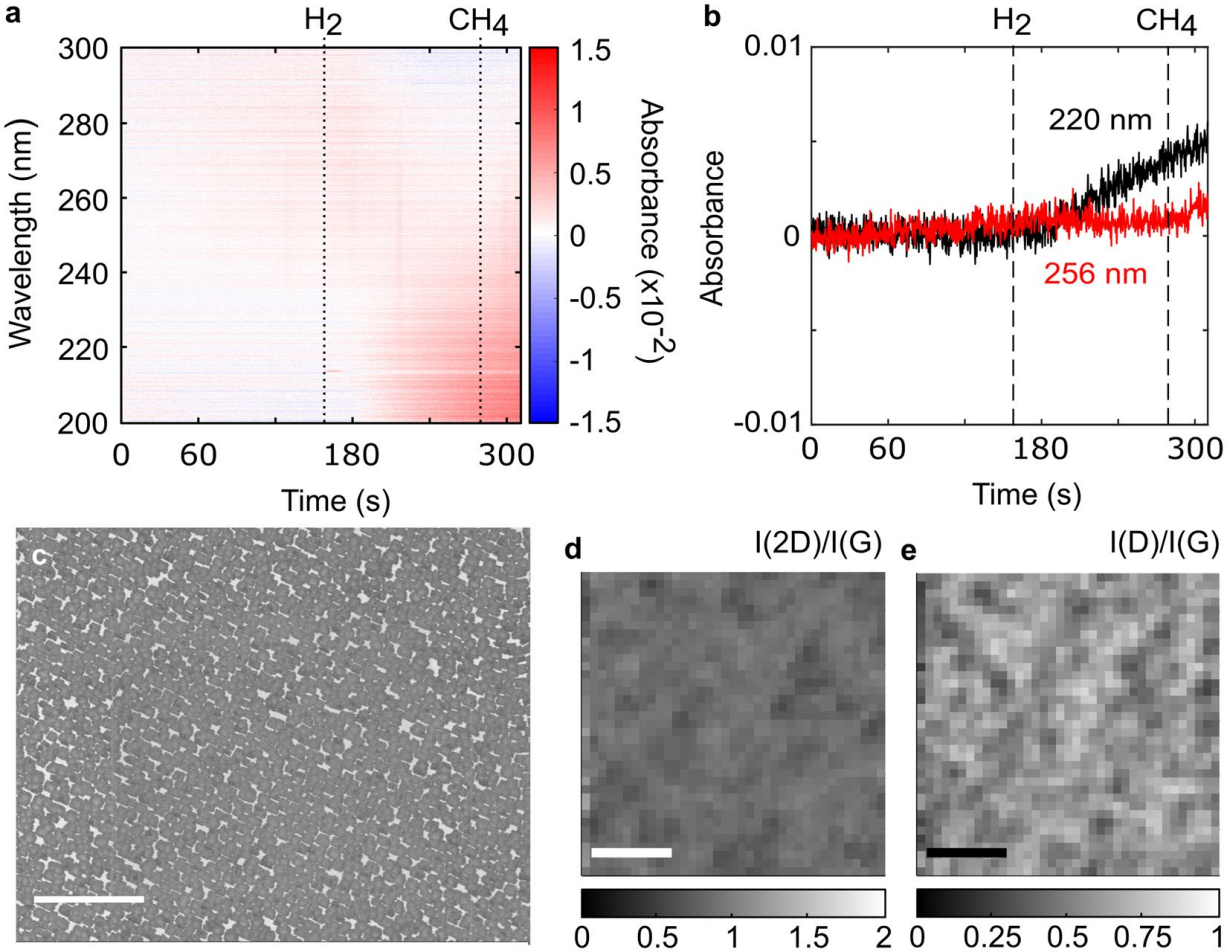
Absorption measurements and characterization of a standard CVD growth when backstreaming is suppressed. (**a**) Time evolution of the absorbance spectra and (**b**) line profiles of the 220 nm (black) and 256 nm absorption peaks (red), with the dotted lines at 158 s and 279 s denoting when H_2_ and CH_4_ are introduced, respectively. (**c**) SEM image of the corresponding growth (scale bar 10 µm). (**d**,**e**) Raman maps of a single graphene grain on copper (**d**) I(2D)/I(G) ratio and (**e**) I(D)/I(G) ratio. Scale bars in (**d**) and (**e**) are 5 µm.

Repeating the growth recipe in Fig. 2 after cleaning of the chamber results in a dramatic change in the absorption spectra and graphene produced (Fig. 5). Negative absorbance peaks are no longer observed when hydrogen is introduced (Fig. 5a,b), and we see a dramatic reduction in multilayer nucleation (Fig. 5c–e) and an increased, more homogeneous monolayer nucleation density. The large I(D)/I(G) ratio in Fig. 5e is in accordance with the large number of edges in the incompletely grown graphene layer. We note that the nucleation density has increased dramatically in this case; we attribute this to the fact that while the exact same recipe was used in both cases, the measured temperature in the reaction zone was ~20–30 °C lower than before. This is likely the result of a change in the thermal reflection characteristics of the chamber upon removal of copper deposits from the chamber surfaces during cleaning. However, we do not expect this to have adversely influenced our findings, since a lower temperature should have increased the occurrence of multilayered nuclei, as reported previously in literature^32^, not reduced it as it has here.

### Characterization of surface contaminants during growth

**Figure 6:**
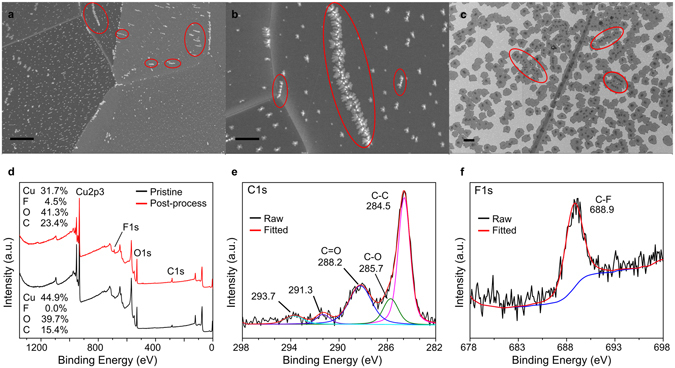
SEM and XPS characterization of a standard CVD growth without methane. (**a**,**b**) SEM images of the copper surface post-growth, showing the morphology of surface contaminants (red circles; scale bars 10 µm and 2 µm, respectively). (**c**) SEM image of a typical growth for comparison, with the highlighted circles showing the morphological similarities between the surface contaminants in (**a**) and (**b**) and the nucleation patterns in (**c**). Scale bar 10 µm. (**d**,**f**) XPS spectra of (**d**) the copper surface before and after growth, with surface elemental compositions indicated for each case (in atomic percent), and of the corresponding (**e**) carbon and (**f**) fluorine signatures.

Figure 6 illustrates the results of repeating the standard growth recipe before chamber cleaning in the absence of methane. White particles with dendritic edges are dispersed across the entire copper surface (Fig. 6a,b) with surface distributions that closely resemble graphene nucleation patterns from similar growths (Fig. 6c). X-ray photoelectron spectroscopy (XPS) of the copper surface before and after growth (Fig. 6d) shows an increase in the carbon concentration and the appearance of fluorine compounds after growth. The C1s spectrum (Fig. 6e) shows, in addition to adventitious carbon, two peaks at 291.3 eV and 293.7 eV. The F1s spectrum (Fig. 6f) shows a clear peak at 688.9 eV that is characteristic of fluorocarbon compounds. While higher energy peaks in the C1s region are more typically associated with plasmon loss features, the fact that graphene should not be present on the surface in this case and that organic fluorine signatures are clearly evident in the F1s region leads us to ascribe the 291.3 eV and 293.7 eV peaks to fluorocarbons instead. Moreover, the peaks correspond closely to the 291.4 eV and 293.8 eV peaks seen for Fomblin^33^, a commonly used perfluoropolyether (PFPE) grease in vacuum pumps, and one that was also present in our pump. We note that organic fluorine compounds are an unusual contaminant to find in electroplated foils inside in a cleanroom; while PTFE containers have previously^34^ been suggested as a potential flourocarbon source, we used polypropylene wafer trays in our experiments, which precludes this as a possibility. The results therefore suggest that the contaminants are introduced during the growth process from the chamber itself, and originate from downstream components such as the pump. Moreover, the striking resemblance between the contaminant distribution and graphene nucleation patterns in Fig. 6a–c indicates that the contaminants were present during the growth phase and actively contributed to nucleation.

## Discussion

Based on our observations, we ascribe the nucleation of multilayers to the products of the reactions of hydrogen with backstreamed carbon contaminants from downstream system components. Backstreaming is a well-known issue affecting vacuum systems, and is especially relevant when operating at low pressures, for extended periods of time, and when using oil pumps^35–40^. Whilst oil-based vacuum pumps can be fitted with oil traps to mitigate backstreaming, a finite amount of backstreaming is almost always present, and the traps themselves can easily be exhausted without regular maintenance^35, 41^. While the results here have been demonstrated for a cold-wall system, similar effects are expected for a hot-wall furnace that is continuously pumped during operation or standby, because pumping at low pressures continuously introduces backstreamed carbon into the chamber (Supplementary Fig. 3). Similarly, while primarily affecting low pressure growths, backstreaming can also affect atmospheric pressure growths if the system is kept in vacuum at room temperature for extended periods of time (Supplementary Fig. 4). Carbon contaminants can also be introduced to the system through other routes and even where dry pumps are employed^41^, such as the condensation in the pumping lines of higher-order hydrocarbons produced during CVD reactions^40^. In such cases, backstreaming also poses a potential issue for dry pumping and turbomolecular pumping systems.

The exact chemical composition of the contaminants is still under investigation; the differences in the rates of change in the 220 nm and 256 nm absorbance peaks suggest that more than one species is likely involved. However, the exact chemical identities and concentrations of the species cannot be determined solely from UV absorption data due to numerous reasons: (1) there is very limited literature on UV absorption spectra of species at elevated temperatures with which to benchmark our results, (2) UV absorbance of most species is temperature-dependant, precluding the use of room temperature data for analysis, (3) the measured absorbance is a convolution of absorption by mutiple species, which makes accurate quantification difficult. As such, a more quantitative technique would be needed to identify the species responsible. Nevertheless, It is well-known that backstreaming contaminants are primarily composed of vapours or degraded fragments of pump oils and greases; these comprise a wide range of lubricants used in vacuum systems, such as fluorocarbons, silicones, olefins, esters, and ethers^36, 39^.

While the XPS results in Fig. 6 certainly implicate fluorocarbons as the culprit, the exact composition of contaminants is likely to be more complex and variable, as the type and concentration of backstreamed species depends strongly on the specifics of the growth system and its operational history^35, 37, 38^. As such, the composition can vary from system to system, and even between runs on the same system. This variability can, for instance, be seen when comparing the hydrogen spectra of Figs 2, 3, 4, and Supplementary Fig. 2. Furthermore, previous reports in literature on hydrogen etching of the quartz walls and subsequent formation SiO_x_ impurities^42^ could equally be explained by backstreaming of silicone-based greases. As such, it is unlikely that a single species plays a dominant role in multilayer nucleation in all cases, but rather a plethora of species that can collectively be categorized as higher-order carbon contaminants.

Backstreamed contaminants may nucleate multilayers by providing an impurity site that locally lowers the chemical potential for graphene growth^43, 44^, but in light of the fact that these are carbonaceous contaminants, we believe our results support a novel explanation for the mechanism of the formation of multialyers during the CVD growth of graphene: hydrogen-induced cracking, volatilization and subsequent deposition of higher-order carbon contaminants onto the copper surface presents a large, instantaneous flux of carbon that triggers premature local supersaturation and simultaneous nucleation of multiple layers. The rapid nucleation and growth that such a large initial carbon flux would furnish would also result in the formation and encapsulation of defective multilayers and residual amorphous carbon at the centre of graphene domains, which we observe via Raman spectroscopic maps (Fig. 2d,e), and a shift from attachment-limited hexagonal growth to diffusion-limited growth of irregular grain boundaries, which we observe via SEM (Fig. 2c). While the broader question of the role of higher-order carbon species present in the gas-phase in multilayer nucleation is the topic of several recent theoretical studies^11, 45–47^ and requires further experimental confirmation, our results provide compelling evidence to suggest that the deposition of higher-order carbonaceous species onto the catalyst surface contributes significantly to multilayer nucleation.

Beyond the initial nucleation, the ultimate size of the secondary graphene layers would be governed by the carbon diffusion length and the rate at which the primary monolayer expands. In principle, growth of multilayers could be sustained through the modulation of the carbon chemical potential (i.e., H_2_ and CH_4_ flow rates), such that the initial monolayer grows fast enough to prevent depletion of excess carbon from the nucleation point (and hence suppress adlayer nucleation), but not so fast that it encapsulates and stunts the growth of the secondary layers. Indeed, this ‘sweet spot’ for CH_4_/H_2_ flowrates has been demonstrated by several studies^20, 23, 24, 48^.

It is important to note at this stage that the absorbance values reported here represent extremely low concentrations of contaminants (on the order of ppm concentrations). As such, the contaminants themselves do not provide sufficient carbon chemical potential to nucleate and sustain the growth of graphene in the absence of methane^18^, which explains why no graphene domains were seen when repeating the growth without methane (Fig. 6).

In conclusion, we have used a novel *in-situ* spectroscopic tool to probe the effect of gas-phase dynamics on the CVD growth of graphene, and have found that carbonaceous contamination introduced into the chamber through vacuum backstreaming plays a key role in multilayer nucleation. This holds important implications for both research into graphene growth dynamics and the large-scale manufacture of graphene^5^, where continuous operation is required, and where oil pumps are frequently used. Moreover, the findings here are also applicable to dry pumping CVD systems, where pyrolytic products have accumulated in downstream components through continuous use, and to atmospheric pressure systems, where the chamber is continuously pumped down while in standby. As such, modifications in operating procedures should be considered to ensure that backstreaming and general carbon contamination in the chamber are minimized. For instance, switching from wet pumping systems to dry pumps, regular maintenance and cleaning of downstream and chamber components (and cleaning components in furnaces), reducing pumping times when there is no gas flow, and operating at atmospheric pressures during standby and operation modes are potential steps that can be taken to mitigate the effects of backstreaming.

More importantly, the findings presented here address the commonly reported observation that the presence of large hydrogen concentrations during graphene CVD, and in particular during low pressure growths, induce multilayer nucleation. In a broader perspective, the results also point to an unexplored route by which multilayer nucleation may be initiated, where the presence of higher-order carbon species in the gas-phase induce premature local supersaturation and nucleation of multilayered domains. Finally, gas-phase UV absorption spectroscopy proves to be a non-invasive and sensitive tool for the *in-situ* study of gas-phase dynamics on graphene growth. In light of its relevance towards preventing multilayer nucleation and monitoring backstreaming in real-time, this method may prove to be a scalable and versatile process monitoring tool for large-scale graphene CVD systems, and for vacuum systems in general where backstreaming poses a potential concern.

## Methods

### Copper substrate preparation

To mitigate the effects of rolling lines and impurities associated with commercial foils, high-purity electroplated copper substrates were fabricated in-house. A thin layer (100 nm) of copper strike layer was sputtered (Lesker, 99.999%) onto RCA-cleaned SiO_2_ wafers in a cleanroom facility. 30 µm of copper was subsequently electroplated onto the strike layer in an acidic electroplating bath consisting of 94 g/L CuSO_4_∙5H_2_O (Sigma Aldrich, ≥98.0%) and 100 ml/L H_2_SO_4_ (Merck, 95–97%). Copper electroplating in a sulfuric acid bath is highly selective towards copper deposition, but nevertheless, low current density DC plating was employed to avoid co-deposition of potential impurities from the solution. The plated films were thoroughly rinsed in deionized water and dried with a combination of acetone and isopropanol rinsing and blow-drying under N_2_ flow. Finally, the foils were delaminated from the SiO_2_ wafer immediately before inserting them into the growth chamber, revealing an atomically flat surface (Supplementary Fig. 5), onto which our growths were subsequently carried out^17, 49, 50^.

### *In-situ* UV absorption spectroscopy

Growth and spectroscopic measurements were conducted using an Aixtron Black Magic cold-wall CVD system situated inside a cleanroom. Figure 1a provides a schematic illustration of the experimental setup. Source gases (Ar, H_2_, CH_4_) are introduced into the chamber via a showerhead assembly, whereby the gases flow past the top heater and heater sample stage, eventually exiting the chamber via the gas outlets at the bottom of the reactor. The reactor uses a rotary vane oil pump (Leybold) and a sorbent foreline trap filled with alumina beads. Two quartz viewports, situated 180° from one another, provide optical access to the chamber (Fig. 1a,b). The optical path length between the two viewports is 80 cm. A deuterium lamp source (Ocean Optics D-2000-S-DUV) transmits deep UV light at an intensity of 3 mW/cm^2^ through one of the viewports (Fig. 1b), and a spectrometer (Ocean Optics QEPro) with a CCD detector tuned to a wavelength range of 200–300 nm collects the transmitted light via the other viewport (Fig. 1b,c). The viewports and the quartz sleeve were replaced with UV-grade quartz for all measurements. Both the light source and the detector are coupled to solarisation-resistant fibre optic cables and UV-grade collimating lenses to project and collect light from the chamber, respectively. The beam is approximately 2 cm above the substrate platform.

A series of transmission spectra are continuously recorded from 1–3 minutes before the end of the annealing phase until the end of the growth phase. All spectra are acquired in single acquisition mode with the same integration time. The integration time was adjusted to be in the range of 800–1000 ms prior to each experiment to obtain the maximum transmitted signal at room temperature while the system is at base pressure. A dark spectrum is taken shortly before the continuous recording, which is then subtracted from all subsequent spectra. The initial spectrum in the spectral series is used as the reference spectrum. Absorption spectra are subsequently calculated for each spectrum in the series using the equation below:

2 $$A={\mathrm{log}}_{10}(\frac{{I}_{0,k}}{{I}_{i,k}})$$

where *I*_0,*k*_ and *I*_*i,k*_ are the spectral intensities of reference spectrum (in this case the initial spectrum intensity) and the *i* ^th^ spectrum, respectively, for a given wavelength, *k*. A series of absorption spectra is thus obtained, with the time interval between spectra defined by the integration time for the experiment.

### Graphene growth

CVD growth of graphene was performed in the Aixtron Black Magic cold-wall CVD system as follows: A small piece of copper foil is placed at the centre of the substrate platform. The chamber is subsequently pumped to base pressure (0.03 mbar for 10 minutes) and flushed with argon (99.9995%, AGA) three times before being pumped down to base pressure for 10 minutes again. The chamber is then pressurized to 20 mbar with argon and heated to 1000 °C (with both the top and bottom graphite heaters on) under 700 sccm argon flow. The copper foil is annealed for 30 minutes at temperature, after which 1000 sccm H_2_ is added. After 2 minutes, once the gas flow has stabilized, 1 sccm CH_4_ (99.9995%, AGA) is added for 30 seconds. The sample is subsequently cooled down under 1000 sccm Argon flow.

### Cleaning of the chamber and downstream components

The chamber was cleaned by disassembling and manually cleaning individual components or replacing them with new ones. Quartz components were replaced with new ones, or were cleaned in a copper etchant solution and annealed in a furnace at 1200 °C to burn off carbon contaminants. Finally, the chamber was heated to 550 °C and an air plasma generated for 15 minutes to burn off any remaining carbon contaminants. This procedure was found to be effective at removing contamination from the system, as judged by spectroscopic measurements (Supplementary Fig. 6). The downstream components were cleaned as follows: the pump was replaced with a new one, the pump hose and filters were cleaned, and the foreline trap was refilled with new alumina sorbent beads.

### Sample characterization

Scanning electron microscopy (SEM) was conducted with a Zeiss LEO 1550 at 5.00 keV. Raman micro-mapping was performed with a Thermo Fisher DXR microscope (455 nm, 6 mW, 10 s × 3 exposure time, 100× objective, 500 nm step size). X-ray photoelectron spectroscopy (XPS) measurements were performed with a Thermo Fisher Scientific K-alpha XPS system using a Al Kα X-ray source (1486.7 eV).

### Data availability

The data that support the findings of this study are available from the corresponding author upon request.

## Electronic supplementary material

Supplementary Information
