# Supplementary material for: Probing the Gas-Phase Dynamics of Graphene Chemical Vapour Deposition using in-situ UV Absorption Spectroscopy
Source: Sci Rep. 2017 Jul 21;7:6183. doi: 10.1038/s41598-017-06276-y (PMC5522467; doi:10.1038/s41598-017-06276-y)
Supplement: Supplementary file 1 — Supplementary Information [file 41598_2017_6276_MOESM1_ESM.pdf]

Supplementary Information

Probing the Gas-Phase Dynamics of Graphene Chemical Vapour Deposition using in-situ UV Absorption Spectroscopy

Abhay Shivayogimath[1], David Mackenzie[1], Birong Luo[1], Ole Hansen[1], Peter Bøggild[1,2], Timothy J. Booth[1]

[1]*DTU Nanotech, Technical University of Denmark, Ørsteds Plads, 345E, Kgs. Lyngby 2800, Denmark.*

[2]*Centre for Nanostructured Graphene (CNG), Technical University of Denmark, Ørsteds Plads, 345C, Kgs. Lyngby 2800, Denmark.*

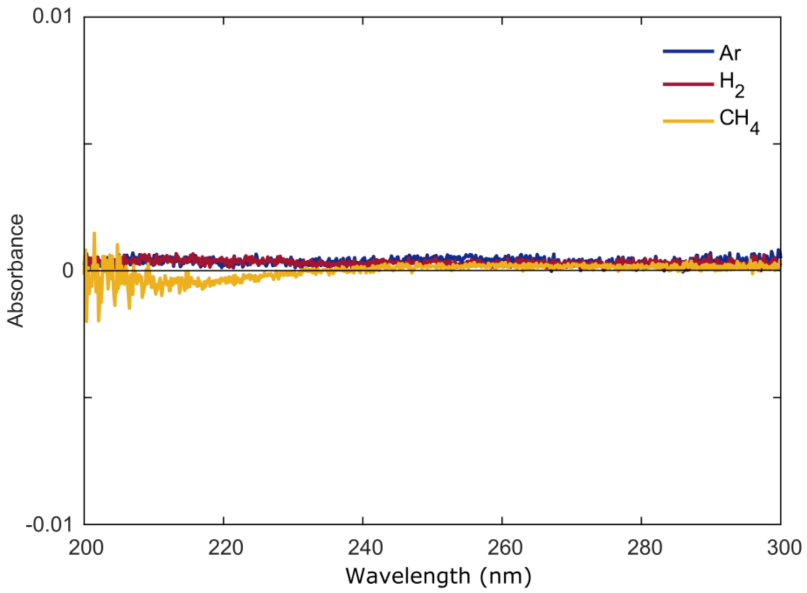

Supplementary Figure 1. Room temperature absorbance measurements of the source gases: argon (blue curve), hydrogen (red curve), and methane (yellow curve).

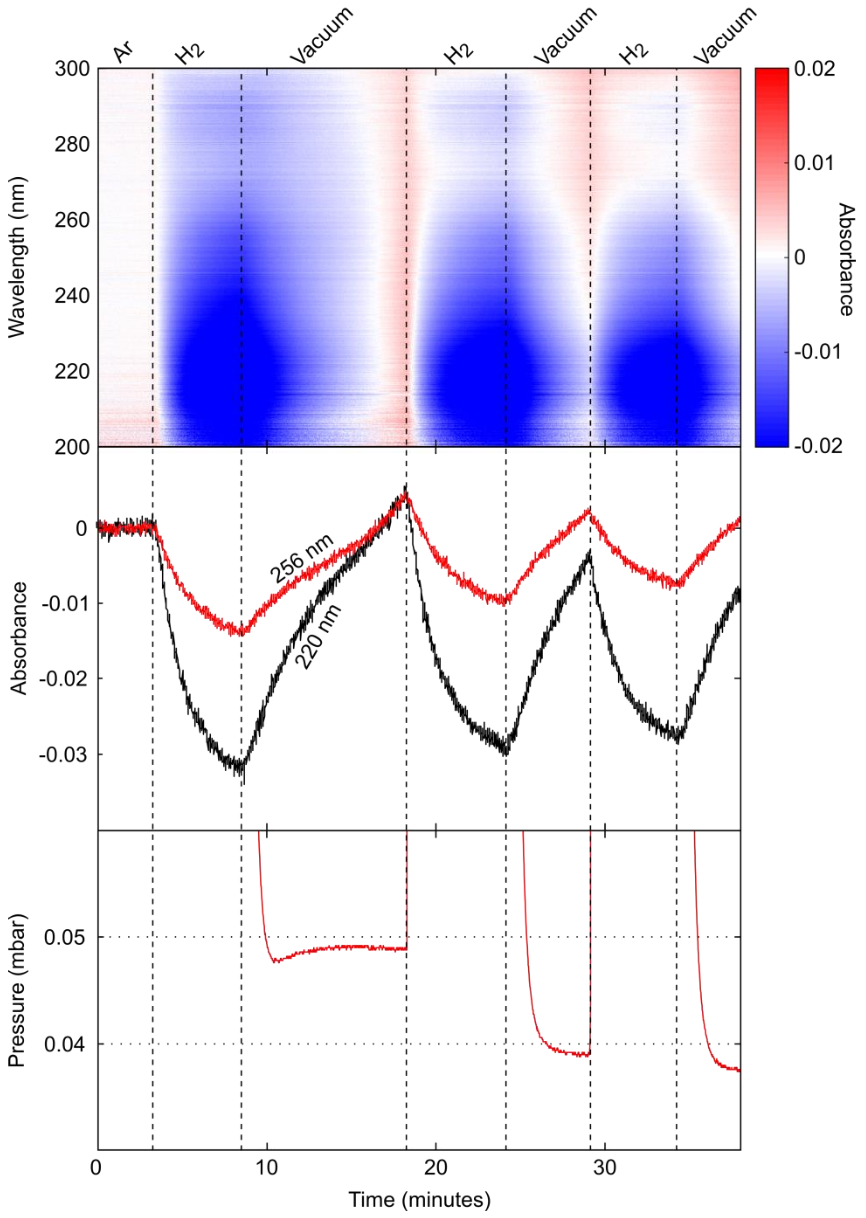

Supplementary Figure 2. Absorbance and pressure changes during H_2/vacuum cycling experiments: (from top to bottom) Absorbance spectra over the entire duration of the experiment, with headers indicating the experimental step; Time evolution of the 220 nm and 256 nm peaks; Base pressure during each pump down cycle.

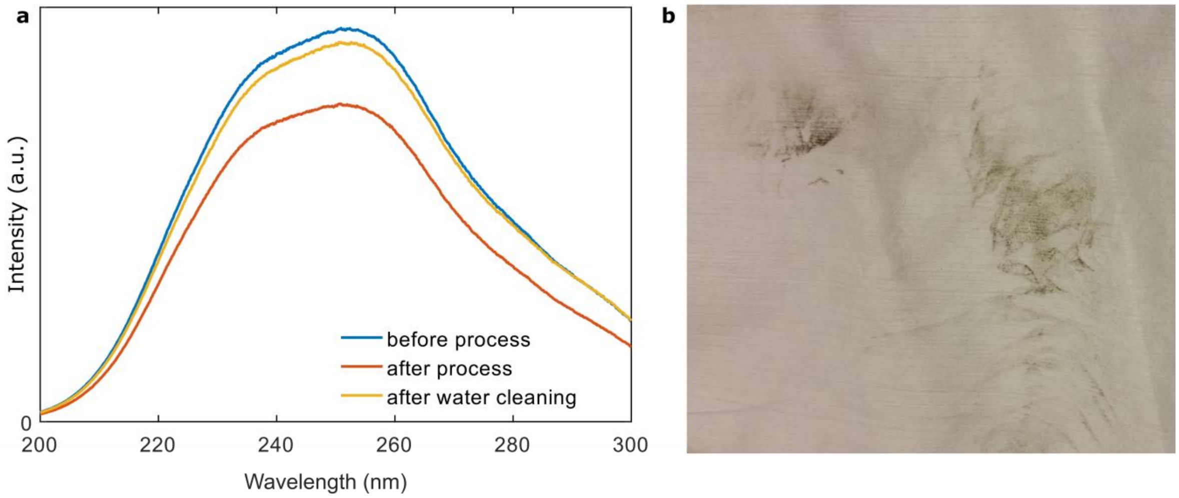

Supplementary Figure 3. Room temperature measurements following H_2/Vacuum cycling experiments: (a) Room temperature transmission spectra before (blue) and after (red) H_2/vacuum cycling experiments, and after cleaning the chamber walls and quartz sleeve with water-soaked lint-free tissue (yellow). **(b)** Sooty deposits seen on the tissue surface.

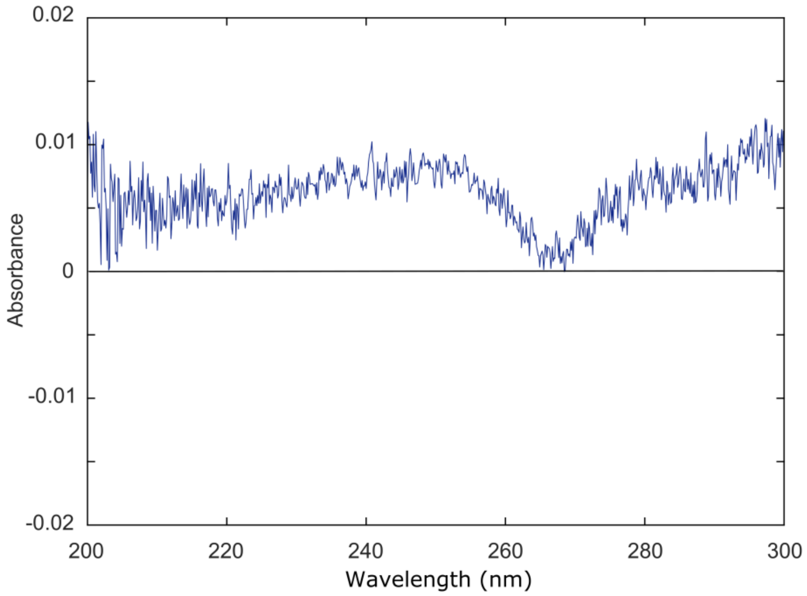

Supplementary Figure 4. Room temperature absorbance after the system is under vacuum over 48 hours: a positive absorbance illustrates an accumulation of contaminants in the chamber with reference to the vacuum spectrum recorded 48 hours earlier.

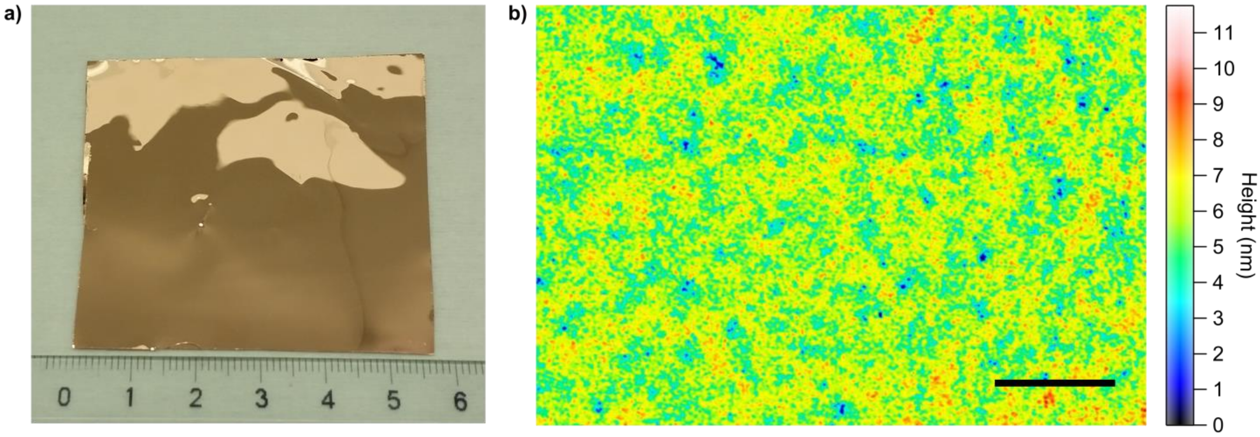

Supplementary Figure 5. Surface roughness of as-peeled copper foils. (a) Optical image of as-peeled foils (scale in cm). **(b)** White light interferometry measurements of the surface roughness of as-peeled copper foils (scale bar 20 µm), with R_q = 1.25 nm and R_a = 0.99 nm.

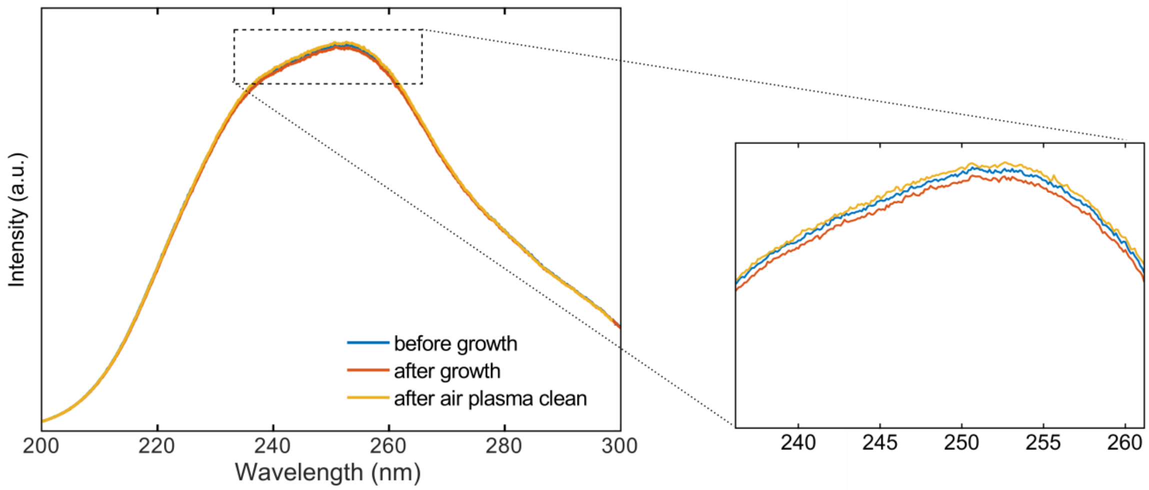

Supplementary Figure 6. Spectral measurements of the efficacy of air plasma cleaning: Room temperature transmission spectra prior to a CVD growth (blue), after a growth (red), and after cleaning the chamber with a high temperature air plasma (yellow). Inset shows a close-up of the peak, illustrating a near complete recovery of the spectra following plasma cleaning.

Supplementary Notes:

H_2/vacuum cycling measurements. To determine the identity and source of the contaminating species, the etching behavior of hydrogen was investigated further. The chamber was cleaned in order to determine whether the contaminants are coming from inside the chamber or from elsewhere in the system. This was done by heating the chamber to 550°C and cleaning with an air plasma for 15 minutes. The chamber was subsequently heated under 700 sccm Ar and 20 mbar to 1000°C, after which 1000 sccm H_2 was introduced. The H_2 flow was ended after a period of time, and the system pumped

down to base pressure. After several minutes in vacuum, 1000 sccm H_2 was again introduced into the chamber, and the system again pumped down. This cycle was repeated once more. No copper substrate was present in the chamber during the experiment. Supplementary figure 2 illustrates the results from this experiment, as well as the associated changes in system base pressure. As is evident, the absorbance decreases everytime hydrogen is introduced, but recovers when the system is pumped down again. The absorbance attenuates less during each subsequent hydrogen cycle. Furthermore, the peak absorbance is lower in each subsequent vacuum phase, which indicates that the chamber is getting cleaner after each phase of introducing hydrogen. The pressure changes in supplementary figure 2 corroborate the spectral data, where the base pressure following each hydrogen cycle improves from 0.05 mbar before the first cycle to 0.038 mbar after the final cycle. Thus, the results indicate that hydrogen is cleaning the chamber of contamination, which re-accumulates inside the reactor when the system is pumped down to low pressures. From this, we conclude that the contaminants originate downstream of the pump valve.

Upon cooling down the chamber, a post-process spectrum was recorded and compared to pre-process spectra to determine whether the aforementioned contaminants remained in the chamber after the process run (Supplementary Fig. 3a). While some recovery in the transmission spectrum was observed, a definite spectral attenuation from pre-process spectra is also evident. This suggests that the contaminants deposit on the chamber walls and accumulate inside the chamber following a process run. Wiping the quart sleeve with water-soaked lint-free tissue (see supplementary methods) reveals dark, sooty, greenish deposits upon the tissue surface (Supplementary Fig. 3b). When a post-clean spectrum

was subsequently recorded, a dramatic reversal of the attenuations was observed (Supplementary Fig. 3a). From this, we deduce that the deposits were directly responsible for the attenuations, and that they are at least partially representative of the contaminants accumulated in the chamber (we note that there are still some differences between the post-cleaning and pre-growth spectra, but these are due to slight changes in the optical transmission as a result of a slight difference in the quartz sleeve position when it was re-inserted after being removed for cleaning).

Supplementary Methods:

Room temperature absorbance measurements. Room temperature absorbance measurements of the source gases were made as follows: The chamber was first purged three times with argon, and subsequently pumped down to base pressure. A reference and dark spectrum were subsequently taken (100 scans to average) while the system was still in vacuum. A source gas was then introduced at prescribed flowrates (100 sccm for Ar or H_2, 5 sccm for CH_4) and the chamber pressurized to 20 mbar. Once the system had reached a stable pressure, the source flow and the pressure control valve were turned off. A sample spectrum was subsequently taken using the same parameters as for the reference and dark spectra. An absorbance spectrum was finally calculated after subtraction of the dark spectra.

Post-process cleaning with lint-free tissue. The quartz sleeve was removed from the chamber and wiped down with water-soaked lint-free tissue three times. The sleeve was subsequently blow-dried with an N_2 gun to thoroughly remove any tissue and water residues. The sleeve was re-inserted into the chamber and the system flushed three times with argon. The system was then pumped down to base pressure.

Spectral investigation of room temperature backstreaming. To determine whether backstreaming also occurs at room temperature, room temperature absorbance measurements were taken while the system was under vacuum over extended periods of time. Here, a reference spectrum was first taken after the system has been flushed and pumped down to base pressure. The system was then kept dormant under vacuum for 48 hours, after which another spectrum was taken under the exact same parameters and conditions as the reference. The system (or the spectroscopic equipment) was not perturbed in any way during these 48 hours. As such, any changes in absorbance must therefore be coming from inside the system itself (i.e. – from backstreaming).

Surface roughness measurements of as-peeled copper foils. Surface roughness measurements were made with the S neox Optical 3D Surface Profiler (Sensofar) at 100X objective and VSI mode. The foil was attached to an SiO_2 wafer prior to measurement in order to flatten the foil.

Spectral determination of plasma cleaning efficacy. A reference transmission spectrum was taken before a standard growth process, while the system was at base pressure and at room temperature, and after the growth, under the same conditions as the reference. The system was subsequently cleaned using the plasma, after which the chamber was cooled to room temperature and pumped to base pressure before taking a post-plasma clean transmission spectrum. The chamber had not been opened between pre-growth and post-growth spectra.
